# Reputation‐Sourced Authority and the Prospect of Unchecked Bureaucratic Power

**DOI:** 10.1111/puar.13281

**Published:** 2020-10-05

**Authors:** Anthony M. Bertelli, Madalina Busuioc

**Affiliations:** ^1^ Pennsylvania State University; ^2^ Leiden University

## Abstract

We explore the democratic implications of a reputational account of bureaucratic authority. While an influential literature has examined the relevance of reputation—and mutual exchange between principals and agents in public organizations generally—the normative implications of these insights have largely escaped scrutiny. We discuss how reputation‐building impacts both the ability and the motivation of principals to oversee administrative policymaking. We argue that reputation‐sourced authority eschews ex ante incentives through the claims‐making and maneuvering of bureaucrats as they develop reputations with audiences. At the same time, it de‐legitimizes ex post oversight because monitoring and compliance must compete both with reputational authority and with resistance from the audiences that are the very sources of such authority.

**Evidence for Practice**

- Reputation‐sourced authority (RSA) can weaken accountability in representative government at the same time that it promotes “good” policymaking.
- When an agency has lots of RSA, politicians are discouraged from oversight of its existing policymaking and from imposing procedural constraints when delegating new tasks.
- Bureaucratic actors with the most RSA have the potential to override the democratic checks placed on their power and to escape accountability.

It is an axiom of representative government that power demands accountability (Kam, Bertelli, and Held 2020; Manin 1997; Powell 2000). Because powers are delegated, and because bureaucratic agents have the ability to exercise discretion, political control is central to understanding public administration. Exploring this problem has spawned a large literature over the past 40 years investigating the extent to which—and under what conditions—representative political institutions are able to exercise control over unelected administrative officials (cf. Aberbach, Putnam, and Rockman 1981; McCubbins and Schwartz 1984). We explore the implications of a recently influential argument about the value of reputation‐sourced authority (RSA) for democratic accountability. In a principal–agent framework, this argument claims that by cultivating a reputation for producing particular policies, agents build enduring authority in governance (Carpenter 2001, 2010), which transforms the relationship between principal and agent into more of a negotiation than a delegation of constrained authority (Carpenter and Krause 2015). While the empirical value of RSA has been established in a variety of contexts (e.g., Busuioc 2016; Etienne 2015; Moffitt 2010), its normative implications remain largely unexplored.

We acknowledge that reputation has many benefits for policymaking; scholars have seen it as a driver of “legitimated vigor” for public bureaucracies (Carpenter 2010, 752), a significant force for state‐building (Carpenter 2010; Carpenter and Krause 2012), and a catalyst for depoliticizing decision‐making (Krause and Corder 2007). Taking the perspective of sustaining representative government rather than enhancing policy quality, we reflect on the implications of RSA for political accountability, specifically that of elected legislative actors in representative governments. This essay aims to serve as a counterbalance to what we call an epistemic view of RSA, which considers its ability to produce “correct” policies. In doing so we do not deny that there are instances in which RSA can yield socially beneficial innovation, and such benefits have been documented empirically (Carpenter 2000, 2001, 2010; Huber 2007). It is also not our intention to defend or to assess critically representative government and electoral accountability against other means for achieving self‐rule, impeded as they are by important considerations ranging from clarity of responsibility (Powell and Whitten 1993) to the overrepresentation of higher income citizens’ interests (e.g., Carnes 2012) to malapportionment (e.g., Samuels and Snyder 2001) to particularistic distributions of policy benefits (e.g., Kriner and Reeves 2015). In this sense, our argument is practice‐based^1^: representative government and its electoral accountability mechanism may well have these problems, but it is constitutionally implemented in the nations in which most RSA scholars situate their work.

We argue that RSA can weaken accountability in representative government for two reasons that, to our knowledge, have not been considered in the literature. First, when an agency has RSA, a political principal's incentives to make and to enforce *ex post controls*, such as monitoring and oversight, are diminished. The agent's reputation alters the political costs of using ex post controls. Second, to generate RSA, bureaucrats make claims to and negotiate with audiences that include political principals, but are not dominated by them (Carpenter and Krause 2012, 2015). Agency success in these coalition‐building endeavors displaces the kind of *ex ante controls* that lie at the heart of political control theories, for instance, statutory procedural requirements (cf. Bertelli and Doherty 2019; Bertelli, Travaglini, and McCann 2019). Essentially, both ex ante and ex post political control compete with RSA, and they may meet resistance from the very audiences that are the sources of RSA.

Scholars taking the RSA perspective have been acutely concerned with its epistemic value for policy outcomes, but are generally silent on the normative implications for accountability arising from the de facto competition between RSA and political control. At the same time, they present RSA as a potential counterweight to problems with political oversight itself. In contrast, we argue that bureaucratic actors with the most RSA risk being exempt from monitoring and oversight, overriding the democratic checks placed on their authority. In other words, power can bypass responsibility. And while the principal–agent conception of bureaucratic authority focuses on a trade‐off between expertise and control, genuine bureaucratic expertise becomes difficult to distinguish from a kind of misperceived expertise, accepted by audiences as the result of bureaucratic claims‐making (Alon‐Barkat and Gilad 2017). Finally, such claims‐making can be directed towards non‐representative audiences that are responsible for building and maintaining an agency's RSA, thus, undermining retrospective accountability through elections. Trade‐offs against democratic values are made frequently in policy domains, and we certainly acknowledge this (Bertelli 2021). Our argument is intended to highlight the need for such trade‐offs to be made clearly and appropriately in the presence of RSA.

We begin our argument by characterizing the costs and benefits of delegation for democracy, after which we illustrate them in the two core elements of RSA: expertise acquisition and audience responsiveness. While acquiring expertise is a well‐established trade‐off in delegation more generally (cf. Bertelli 2012; Gailmard and Patty 2013; Ting 2011), responsiveness to audiences is the gravamen of our contention, and it commands the bulk of our attention. Our essay culminates in a call for further theoretical and empirical consideration of the potential adverse effects of bureaucratic reputation for accountability.

## The Democratic Costs and Benefits of Bureaucratic Expertise

We begin our discussion by fixing terms about representative government so that we can clearly account for the costs and benefits that RSA presents to it. With Powell (2000, 3), we address “the claim of democracies to be governments in which the people participate in policymaking.” Moreover, “elections are instruments of democracy to the degree that they give the people influence over policy making [and] such citizen influence is a good thing…elections should not only provide symbolic reassurance” (3–4). *Democratic costs* are reductions in the extent to which the people participate in making policy, while *democratic benefits* are increases in such participation. We must be quick to note that as with elections, we believe that participation in democracy must be representative. As a consequence, advisory committees and other non‐representative mini‐publics cannot take the place of elections regardless of the clarity of electoral expressions of interest (e.g., Moffitt 2014).

Delegation to bureaucracy has costs and benefits to a political principal that scholars have considered carefully. Its benefits are effort reduction (Bendor and Meirowitz 2004), expertise acquisition (Gailmard and Patty 2013), uncertainty reduction (Bendor, Taylor, and Van Gaalen 1987), special interest protection (McCubbins, Noll, and Weingast 1987), and blame shifting (Fiorina 1986). The costs to political principals come in the form of agency loss—that is, policy conflict—information asymmetries, and capacity deficits (see Bertelli 2012, 80–91).

To mitigate agency loss, political principals can provide for *ex post controls* in the form of monitoring—ex post review of agency actions—and oversight provisions (see generally Bertelli 2012, ch. 3). Yet weighing the costs and benefits is not always clear cut: expertise acquisition is a case in point. A recent delegation literature provides important insights about the ability of the agent to shape the terms of the formal contract through *expertise investments*, which differentiate political principals, who are policy generalists, from their bureaucratic agents, whose expertise makes them specialists (Callander 2008; Gailmard and Patty 2007, 2013; Ting 2011).

The endogenous development of bureaucratic expertise and capacity^2^—the agent's ability to control and to vary the supply of expertise, rather than to work with a fixed and exogenous supply of expertise—allows agents to shape the principal's incentives. Bureaucrats can, for instance, strategically invest in “targeted capacity” to implement policy, improving their ability to carry out a governance task while constraining the principal's policy options (Ting 2011). Essentially, specialized capacity investments increase the agent's bargaining power. In generic principal–agent problems, this might be a good thing—high‐powered incentives from the principal have the ability to crowd‐out a mutually beneficial trust (Miller and Whitford 2002). But, as we will explain, we believe that there are democratic costs stemming from agent authority in the particular problem of bureaucratic agency that cannot be easily dismissed.

Because of its ability to shape the principal's incentives, expertise acquisition comes with democratic benefits and costs as we have just defined them. Table 1 depicts these costs and benefits as they relate to the core components of both expertise‐sourced authority (acquisition of expertise) and RSA (responsiveness to audiences). Regimes of costs and benefits due to these core components are depicted in the principal quadrants of the table and discussed below. The principal democratic benefits of expertise acquisition accrue in an (ideal) regime of *expert delegation*. This regime prevails when bureaucrats acquire real expertise that is germane to agency policymaking and, subject to the ex ante and ex post mechanisms of political control, use it to serve the political principal's policy interests. Expert delegation allows ideologically different agents^3^ to serve the principal's aims, and for those aims to be retrospectively held accountable in elections (Powell 2000).

**Table 1:** Expertise‐sourced versus reputation‐sourced authority

	Democratic benefits	Democratic costs
Expertise acquisition	Expert delegation	Strategic capacity‐building
Audience responsiveness	Alignment of expertise and perception	Misperception (undeserved reputation)
	Representative audiences	Diminished incentives for oversight
		Principal override

The top right quadrant describes a regime that diverges from this ideal. When bureaucrats and politicians differ in their policy preferences, bureaucrats have the incentive to acquire expertise in a manner that incentivizes politicians to weight the informational benefit of delegation more heavily than the distributional benefit. For instance, but for the expertise that agency A acquired, the policy conflict between politician P and A would have been too great for P to delegate authority to A. This regime of *strategic capacity‐building* prevails when “agencies can strategically acquire expertise as a means of setting the policy agenda” (Bertelli 2012, 128).^4^ The principal can be faced with a trade‐off between inferior implementation of its desired policy or implementation of a policy that is further removed from its ideal (Ting 2011). Gailmard and Patty (2013, 2) note that such control sacrifices are normatively justifiable as the agent brings greater information to bear on public policy: “for all the accountability problems it presents, the value of high quality information cannot be seriously doubted. (…) we must reconcile ourselves to institutional forms that leverage the information necessary for policymaking through the power to act on it.” Again, “[a]lthough such an approach sacrifices control over policy, its upside is better information in the bureaucracy” (Gailmard and Patty 2013, 7). Strategic capacity‐building thus forces a trade‐off between policy conflict and informed policymaking that can work to bureaucrats’ advantage.^5^

We are more wary of the democratic costs to the policy agenda and the capacity for legislators to represent their constituents. Even the most ardent advocates of delegate representation believe that the information that is transferred to a political principal from the ballot box is limited. Christiano (1996) argues that this information can constitute a set of policymaking priorities and trade‐offs among them. In this way, the party manifesto in parliamentary systems commits—though it does not legally obligate (Manin 1997)—a party to a schedule of priorities and trade‐offs that they would assume in governing. Strategic capacity‐building can have the effect of removing priorities from, adding priorities to, or substituting priorities within this schedule.^6^ While we agree with Miller and Whitford (2016, 130) that repeated interaction can create a mutual check on principal and agent that can lead to the enforcement of a norm of compliance, the informational advantage of the bureaucrat allows strategic capacity‐building to *increase* with repeated interaction and to allow the bureaucrat to make priorities and trade‐offs, instead of the electorate. The benevolent norm of trust that can form repeated interaction in general agency relationships has a different meaning in representative government.

Our discussion of the democratic costs and benefits of *audience responsiveness* in the bottom quadrants of Table 1 is distinguishable from an *epistemic* view of RSA that prevails in the seminal literature. This view has two elements. First, accountability to representative government can produce maladministration. This is because the preferences of elected politicians, whether or not they are induced by the wants of the electorate, will not (or have not) achieved a “correct” policy. Second, RSA helps to remedy this situation in the long game. Over time and through experience with changing states of the world, an epistemic conception of RSA allows bureaucrats to become better at making “correct” policies, and they can use their reputation with audiences to maintain them.^7^ We characterize this view as epistemic because it focuses on some objectively possible standard of “correct” policy, and has this in common with epistemic theories of democracy (cf. Cohen 1986; Estlund 2009).^8^ The epistemic view allows bureaucrats to learn how to make “correct” policy by autonomously making policy, that is, by engaging in the very activity that gives them RSA.

While we do not disagree as an empirical matter that RSA produces policy benefits (cf. Carpenter 2001, 2010; Huber 2007; Miller and Whitford 2016), we seek to uncover the ways in which it challenges representative government, rendering our argument quite procedural in nature. We contend that representative government sets up an accountability relationship and that RSA is “good” if it facilitates accountability between representatives and citizens. Like Bertelli and Lynn (2006, 146), we see do not see accountability “as an ideal for regimes or governments so much as it is an ideal for the individuals who serve in official capacities.” In this way, we claim that accountability is indeed an impediment to the epistemic benefits of RSA, but also to the democratic costs it can impose. We contend that RSA scholars must confront a trade‐off between epistemic value and democratic cost. The RSA literature has emphasized the epistemic view, while we seek to bring more balance by emphasizing a view of representative government that permits entrusting the *means* of policymaking to bureaucrats.^9^ The democratic costs can be assessed only retrospectively in elections, and in what follows, we address the problems that RSA can create for holding administrators and elected politicians accountable for their choices and actions.

## Blunting Political Controls through Reputation

A reputational account, when followed to its logical conclusion from an oversight perspective, much more seriously problematizes this picture. The reputation literature intimates that the successful cultivation of a positive reputation endows organizations with authority (Carpenter and Krause 2015) or, in the language of this literature, with “power” (Carpenter 2010). Audiences—“coalitions of esteem” (Carpenter 2000, 124)—empower organizations beyond their formal statutory authority. Audiences can be both formal and informal, ranging from political actors, to professional organizations, industry actors, or even the media. They are “multiple and diverse” in nature, as opposed to a single clientele, which would amount to capture rather than indicate public‐spiritedness (Carpenter 2001, 32). While discretion is defined by the principal through formal provisions, autonomy is generated outside the statute.

Audience support can empower an organization vis‐à‐vis its principal (Busuioc and Lodge 2016). That is, audiences can emancipate agents from a principal's control mechanisms. Carpenter (2001) recounts that “[b]ureaucratic autonomy prevails when a politically differentiated agency takes self‐consistent action that neither politicians nor organized interests prefer but that they either cannot or will not overturn or constrain in the future” (17). He notes that politicians will defer to bureaucracies with opposing goals if: “(1) failure to do so would forfeit the publicly recognized benefits of agency capacity and/or (2) the agency can build coalitions around its innovations that make it costly for politicians to resist them” (17). So constructed, RSA is a substitute for political oversight under the two foregoing conditions.

The former—a principal's deference to bureaucratic experts with opposing goals in order to reap the benefits stemming from agency capacity—is consistent with the formal literature discussed above that treats expertise as endogenous to delegation (Callander 2008; Gailmard and Patty 2007, 2013; Ting 2011). The latter— that is principal deference as a result of the agent's successful coalition‐building, making it costly for the principal to reject agent claims about what should be done in a policy domain—is specific to reputation literature and recognizes that authority is socially embedded. Both principal and agent are situated in broader networks of audiences. Authority is granted (or withheld) by these networks of audiences, which can enhance agent standing vis‐à‐vis the principal and effectively alter the terms of the formal delegation. The relationship envisaged by principal–agent theories becomes “transactional” (Carpenter and Krause 2015). Authority is not controlled ex ante or exogenously produced, but, rather, redefined in principal–agent interactions and through the intervening role of audiences. Audiences can throw their weight behind the agent, displacing the statutory balance of power. In this vein, Krause and Corder (2007, 131) argue with evidence that agencies with lower personnel turnover and autonomy are less likely to accommodate political influence when applying their expertise than agencies that are less “stable” in these parameters.

In the case of the US Post Office, Carpenter (2000, 122) notes how a strong reputation among “a network of support that spanned party boundaries meant that national politicians were powerless to break apart such a coalition;” “the ‘autonomy’ of agencies in the early twentieth century was…the ability to inaugurate and lobby for programs that elected officials had not imagined and did not initially welcome” (125). Audience support “displaced the logic of delegation. Delegation now became bargaining. The agent became a co‐equal player in national policy battles” (Carpenter 2000, 125). Such displacement contributed to shifting the legitimating narrative from a legislative‐centered argument about detailed delegations reviewed by courts to a bureau‐centered account that proved difficult for administrative law to allow (Bertelli and Lynn 2006, 85–86). Horwitz (1992, 333) writes that the post‐war “declining faith in the ability of experts to produce scientific, neutral, and apolitical solutions to social and legal questions” ushered in an era of formalism in administrative law that expanded the scope of judicial review. Over time, it became clear that understanding who benefits from administrative action would predict who would support greater bureaucratic autonomy (Horwitz 1992; Bertelli and Lynn 2006, ch. 4).

RSA fundamentally re‐draws the boundaries identified through delegation and the crafting of enabling legislation. On the one hand, RSA carries potential democratic benefits through the intervening role of representative audiences (Table 1). Organizational power is imparted by multiple, formal and informal audiences (“coalitional ties”) as opposed to only a political principal motivated by electoral rewards. Mutatis mutandis, enhanced audience responsiveness can protect a pluralist landscape of special interests. If audiences are representative—and in our opinion, this is the key— RSA can have democratic benefits. Such a regime of representative audiences from Table 1 can improve policymaking while respecting representative government.

On the other hand, a new component of the bureaucratic agency problem simultaneously emerges; audience support generates asymmetries of power in the traditional principal–agent relationship—it can feed power imbalances between the elected principal and its agent, favoring the latter. Compared with traditional delegation accounts, RSA comes with democratic costs in regimes of diminished incentives for oversight and principal override in the bottom row of Table 1. RSA can blunt both ex ante and ex post controls, and, crucially, it can do so in a context of enhanced agent autonomy.

Before discussing these democratic costs and benefits in more depth, we note that for us, the principal source of the democratic problems of RSA comes from the ability of the agency to make specific kinds of claims. Our argument is rooted in Saward's (2006) notion of the representative claim, which he believes to lie at the heart of representation and which, when made by agencies in our context, competes with the expression of the electorate in representative government (Manin 1997). The representative claim is not limited to elected officials; cannot be “redeemed” in full and contains ambiguities that make representation itself a set of “claims and their receptions;” is highly contextual; and itself “plays a key role in constituting constituencies (or audiences)” (Saward 2006, 306).

Two styles of representative claim are crucial to the democratic assessment of RSA: a *framing claim* representing expertise to political principals and an *implicit–explicit* claim representing themselves to external audiences as representatives or champions of their interests. The framing claim “delimits and defines the contours of the basic system and constitutionalizes or ‘encodes’ it,” allowing the claims‐maker to take strategic advantage of that scheme when confronting the audience (Saward 2006, 307). Under RSA, this type of claim forms the basis for transactional authority that agents develop with respect to their political principals. Such claims rely on expertise, speaking from the particular to the general (307), and allow bureaucrats to represent aspects of their expertise to political principals in strategic ways as our discussion of strategic capacity‐building suggests. Such claims in our context would take the following form: “the policy environment is X and this agency possesses *unique* capability Z that will yield outcome Y.” The crux of the agency's representational claim is that Z is essential to get Y when X prevails (expertise and perception are aligned) and that Y is a good outcome for political principals (outcome represents the principal's interest).

The implicit–explicit representative claim is made possible by the cultivation of audiences by bureaucrats. The latter “set themselves up,” implicitly or explicitly, as advocates and representatives to the former. Saward (2006, 308) notes that “the style or focus of the claim is familiar, and invokes or rests upon accepted representational…codes” but never entirely drawing upon them. His example is a member of parliament claiming to represent the embodiment of constituency interests on a particular subject (308). Similar claims, we argue, allow the bureaucrat to assure his or her audiences that their interests are being represented in transactions with political principals. In our context, such claims would take the form: “the members of audience A know that when the policy environment is X, we possess capability Z that will yield the outcome Y they desire.” In Saward's words (206, 308), the agent presents itself “as the embodiment of constituency interests (…) to that constituency (audience).” This would be supplemented by subsequent claims‐making to the political principal that the agent is the embodiment of preferences that have the backing of electorally relevant audiences (e.g., swing groups, etc.). The essence of the agency's representation is that Z produces Y, where X prevails (expertise and perception are aligned), and A prefers or should prefer Y (outcome represents audience interest).

## Diminished Incentives for Oversight

In a context of audiences and RSA, the principal's ability to control agent action is impacted—for instance, by delegitimizing action against an agent highly regarded by particular audiences. In addition, the principal's motivation to engage in oversight falls because of the diminished political benefits that oversight brings. In other words, oversight of RSA comes with the potential for high costs and low political payoffs. Furthermore, even in the scenario where oversight activities do take place because politicians are willing to pay these costs and where oversight is effective in the sense that it gives politicians accurate information about an agency's activity, RSA can insulate the agency from the impact of this information through processes of claims‐making to its coalition of esteem.

Although principals might not prefer the outcomes enacted by their bureaucratic agents, they “will not overturn and constrain” these because of the political costs involved in doing so (see Carpenter 2001). In other words, in a reputational understanding of bureaucratic power, the power of the agent will restrict the scope of action of the principal to enact controls on that agent (see also Carpenter and Krause 2015). The enactment of sanctions by political principals towards a highly reputed actor could well come to be perceived as an illegitimate activity by audiences (Busuioc and Lodge 2017). For instance, a principal might well anticipate that sanctioning an agency held in high regard by external audiences will result in audience criticisms and backlash against the principal, preempting such action.^10^ Simply put, the agent's reputation alters the political costs of using ex post controls. These insights are consistent for instance, with the findings of Bertelli and Sinclair (2015) on agency termination, where audience effects, captured by media attention, impact the use of formal controls, the extreme measure of agency termination in this case: “Enhanced media attention can disrupt the normal views politicians have about what an administrative agency does (…) attention in outlets serving the core voters of the parties in government places agencies in less peril of reform” (855).

Influential work has shown that electoral incentives drive oversight (McCubbins and Schwartz 1984; Zegart 2011). Members of Congress have incentives to engage in “fire alarm” rather than “police patrol oversight,” expending resources on oversight only when aggrieved parties sound the alarm (McCubbins and Schwartz 1984). Explains Zegart (2011): “legislators devote their energies to issues that are considered most important to their most important constituencies,” which are crucial to their reelection. This is implicitly regarded as a positive state of affairs as it ensures responsiveness to constituency grievances, as well as efficient and effective oversight as it allows for targeted oversight of actual agent misbehavior and, importantly, of misbehavior that constituents care about. A corollary, demonstrable for intelligence oversight, is that: “when electoral incentives are weak,” “control…will be weak too” (Zegart 2011, 52). Characterized by low voter attention and weak interest groups—and consequently weak electoral incentives—intelligence is subject to restricted oversight by Members of Congress: “intelligence oversight does not pay off.” Zegart continues, “[i]ronically, the type of incentives that foster responsiveness in some areas lead to neglect in others. Intelligence oversight does not tick because there are no electoral incentives” (12).

Given that reputations are embedded in coalitions of support, then oversight and/or sanctioning of such agents would lack political payoff or even more, as noted above, actually incur political costs. In other words, with oversight being a matter of incentives (McCubbins and Schwartz 1984), an esteemed agent reputation among a broad network of support will dampen the principal's electoral incentives for oversight. A reputational lens, then, suggests that the relationship between power and accountability is not necessarily symbiotic. A strong reputation, the very source of organizational power in an RSA narrative, can blunt formal controls.^11^ What is more, it is precisely with respect to powerful organizations that institutional controls can fail. In a context of audience networks and reputation, enhanced agent standing^12^ and authority can decrease the democratic benefits of ex post controls, and with incentives failing to shape their responsiveness to political preferences, bureaucratic power can emerge in unpredictable ways.

## Representative Audiences and Undeserved Reputation

Diminished incentives for oversight are less problematic when framing claims about agency expertise are accurate and less still when implicit–explicit claims are broadly representative. In such cases, it is unlikely that the “fire alarms” of oversight would be sounded and oversight exercised because the agency is a benevolent, high‐capacity organization held in high regard by external and representative coalitions of esteem. In such a scenario, reputation can conceivably function as a heuristic for political overseers in regard to actual bureaucratic performance. In a context of growing “accountability overloads” (Bovens, Schillemans, and Hart 2008) in the public sector, under this scenario, reputation can be a helpful proxy for better targeting oversight.

Reputation however, is “observed capacity” (Carpenter 2000, 125)—it is a matter of perception and presentation of actual capacity: “Complex organizations are seen ‘through the glass but dimly’ by their manifold audiences” (Carpenter and Krause 2012, 27) and ambiguity is crucial to effectively managing different—and potentially conflicting—audience expectations: “what one audience sees is not necessarily what another audience sees…. In part because perceptions differ across audiences so does judgement” (Carpenter 2010, 34). The framing claims of bureaucrats are most influential for this very reason.

In a context where a strong reputation (“audience esteem”) diminishes incentives for political monitoring and oversight, the prospect of undetected mismatches between perceived and actual expertise increases, especially under conditions of greater ambiguity (see Alon‐Barkat and Gilad 2017). In other words, RSA becomes especially problematic because it diminishes incentives for monitoring. Authority is granted on the basis of *perceptions* of expertise and capacity—as opposed to what agents actually possess—in a context of diminished incentives to monitor actual performance. That is, this happens in an environment where principals are processing framing claims about expertise, influencing their ability to ascertain whether perceptions of capacity correspond to reality. Unlike arguments about endogenous expertise, where a principal's trade‐offs are purposive and rational—its sacrifices on control are justified in theory by concrete gains from actual expertise acquisition—a reputation perspective reveals a much more complex picture. Sacrifices on control might not necessarily have been weighed against true increases in bureaucratic capacity and expertise. The compounded effect of misperceiving agency capacity and diminished oversight raises the specter of agency losses and accountability deficits. That is, *undeserved reputation* that goes unchecked is a non‐ignorable cost to representative government.^13^

Such mismatches between audience perception and actual capacity do arise. For instance, Alon‐Barkat and Gilad (2017) find experimentally that citizen exposure to familiar symbols enhances citizens’ positive attitudes towards public organizations and can compensate for their experience with poor bureaucratic performance. Symbolic communications, they find, can enable bureaucracies to maintain positive reputations despite poor performative capacities.

Prior to the financial crisis, the Financial Services Authority (FSA), the UK's now defunct banking regulator, was held in high regard by government, industry, and international regulators alike. As a leading authority on “principles‐based regulation,”^14^ the FSA could claim a model approach to financial regulation emulated internationally (Black 2008).^15^ Enter the financial crisis, which discredited the agency's previously heralded “light touch” approach. Having too light a touch condemned the FSA, in the aftermath of the crisis, as partially responsible for the financial meltdown, with mounting evidence of massively underestimated risks. The “model regulator” went on to be abolished for its failings. In its “obituary” on the FSA, the *Guardian* described it as:

[T]he watchdog that didn't bark. (…) it is likely that it will be remembered for only one thing: presiding over the near‐meltdown of the UK's banking system. In its short life, the FSA failed to rein in the banks, and even encouraged the City to explode in the mid‐2000s with a ‘light touch’ approach to regulation. It did not notice that Northern Rock was built on such shaky foundations that it could easily run out of money, and failed to prevent the takeover of ABN Amro by RBS just as the credit crunch was biting in late 2007.^16^

Yet “[o]nly a year ago it seemed that ‘principles‐based’ regulation was the answer that all policy makers and those running financial institutions had been looking for” (Black 2008, 425).

Bureaucratic cases of the emperor's new clothes occur, and agencies can lose the RSA they have built as a result of policy choices that work against their perceived expertise. RSA can be weakened as a result of this kind of learning. By contrast, networks of formal and informal audiences can also sustain and reinforce misperceptions of organizational image. Organizations are well performing in the eyes of networks of audiences until their expertise is shown to be undeserved and their reputations crumble. Such false confidence is a result of the persuasive value of framing claims. In the FSA case, it took a major financial crisis to dispel previously held (mis)perceptions of high capacity. Similar dynamics are becoming visible with the FDA as well: “The Administration's image has been badly withered in recent years”, notes Carpenter (2010, 748) at the end of *Reputation and Power*. Regulatory scandals, complaints from top scientists about interference with scientific work (e.g., as expressed through the Union of Concerned Scientists survey), of expert advice being overlooked, and low scores on public opinion polls are calling into question the accuracy of its prior image.

Moffitt's (2010, 2014) study on the FDA's voluntary use of public advisory committees in its approval of new drugs provides a revealing example as to the problematic dynamics that can emerge between regulatory image and accountability. While Moffitt (2010) finds evidence of active reputation management by the FDA in its reliance on public advisory committees, what is particularly interesting to note in our present context is that public stakeholder engagement is associated with a reduced prospect of actual oversight: “FDA advisory committee consultations are systematically associated with a chief concern among reputation‐minded bureaucrats: avoiding a Congressional oversight hearing at which the bureaucrats must publicly defend and explain ostensible agency failures. Even though public advisory committees review the riskiest drugs, and those drugs are more likely to go on to experience subsequent safety related problems, the FDA is less likely to find itself on the Congressional hot‐seat when it publicly consults its advisors” (889).

In other words, despite a problematic record of actual performance,^17^ the FDA is less likely to be held to account over its performance in these cases. What is more, it is important to remember that this also occurs in a context where reputational forces lead to the *irreversibility* of agency decisions (Carpenter 2010, 67–68), that is, where the bureaucracy is less likely to police or check itself for erroneous decisions due to reputational costs arising from reversal. In other words, once a decision is made, concerns with reputational damage make retractions and course reversals less likely. As Ferejohn (2012, 799) puts it:

“After subjecting firms to extremely exacting and costly procedures prior to investigating or marketing new molecules, the FDA has been very reluctant institutionally to pull drugs off the market on the basis of postapproval clinical experience.(…) once a firm has gotten a drug through the regulatory gauntlet to the promised land of patent‐protected marketing, it has in effect real money in the bank that is not likely to disappear (short of an outright catastrophe; and Carpenter shows that the bureaucratic routines inside the agency make it hard for a catastrophe to be recognized).”

This argument is also lent support by Potter (2019, 5, emphasis added), who contends that bureaucratic agencies can use administrative tools strategically such as expanding stakeholder participation “to strategically and systematically insulate their rule‐making proposals *from scrutiny and interference*.” Agencies restrict or expand “the participation valve” depending on the political environment they face and the resistance they anticipate:

“[I] n circumstances where groups are likely to bolster the agency's position, the agency may find itself increasing participation opportunities. That is, if groups support the agency's position and principals do not, having groups weigh in on the agency's proposal may yield the substantive benefits discussed earlier and serve to convince overseers that the agency proposal has merit—*or at least that intervening in the rulemaking may raise issues with a groups of powerful interests*” (78, emphasis added).^18^

Audience support makes a principal's intervention costly.

## Principal Override and Coalitional Drift

To allay accountability concerns, one could point at the simultaneously disciplining role of audiences. Reputation has both an emancipating and a constraining effect on public organizations. A reputational perspective would intimate that organizations are not free agents but shape their behavior in response to audience expectations. Thus, there is representation and its attendant responsiveness, but to audiences rather than principals and supported by the strength of implicit–explicit claims to audiences that the bureaucrats are indeed representing them. Moreover, reputation requires a multiplicity of cross‐cutting ties in terms of audience support. The linchpin is the implicit–explicit claim that audiences are being represented just as effectively as through elected representatives: “autonomous agencies…shift electoral and representative preferences” (Carpenter 2001, 357). In other words, while reputation can amount to a principal override, principals are *persuaded* to adapt their preferences by framing claims of expertise in light of audience costs which have political manifestations: “principals find it in their long‐term interests to defer” (Balla 2003, 106). It is, in fact, on this very basis that RSA is legitimized: resulting agent autonomy is not illegitimate (a form of bureaucratic drift) as political actors come to shift their preferences.

In our view, in its generalization of audiences (“cross‐cutting ties”), the bureaucratic reputation literature sidesteps the normative problem associated with non‐representative audiences. As noted above, theoretically, RSA can have potential democratic benefits insofar as audiences are broadly representative of the public at large. A representative audience regime would prevail when audiences and voters are more closely aligned than politicians and voters. This narrow possibility is the only situation in which RSA provides democratic benefits under representative government. Only then would the implicit–explicit claims‐making of bureaucrats justify their empirically observed actions to contravene principals’ interests (Potter 2019).

On the contrary, principal override effectively amounts to audiences constraining or shifting the preferences of democratic and constitutional principals and has the potential to seriously compromise democratic responsiveness to “the wants of the public as expressed by the public” (Finer 1941, 337; but see Christiano 1996). What results, we argue, is a manifestation of the other type of “drift” the political control literature warns against: “legislative or coalitional drift” (Horn and Shepsle 1989; Shepsle 1992). Horn and Shepsle (1989) argue that in addition to bureaucratic drift, principals are concerned about coalitional drift, that is, about the composition of future policy coalitions and preventing them from tampering with the terms of the enacted agreement. Writes Shepsle (1992, 114), “the enacting coalition in the statute game must, at the time of enactment, worry not only about bureaucratic discretion (…) they must also consider the prospect of subsequent plays of the statute game.” General ex ante controls such as administrative procedures are set up to mirror the coalitions of interests that gave rise to the agency's legislative mandate as a way to ensure compliance with the will of the enacting legislative coalition and the durability of their agreement (McCubbins, Noll, and Weingast 1987).

Seen from this perspective, RSA and the implicit–explicit claims of bureaucrats constitute a mechanism through which agencies can build new coalitions around their programs to shift those of enacting coalitions. In other words, reputation‐building can become a mechanism through which strategic bureaucratic agents can *cultivate coalitional drift*, potentially reversing the inverse relationship between bureaucratic and coalitional drift described in the political control literature.^19^ Bureaucratic and coalitional drift thus come to co‐exist, with the former working through the latter. The agency can take preference‐consistent actions by forging new coalitions around the policies it prefers, exerting pressure on its political principals to adapt their preferences ex post. Politicians can become persuaded through bureaucratic claims‐making to abandon an enacting coalition's position. What results is an intertemporal divergence in political preferences—democratically ambiguous because it may at times yield outcomes closer to current principals and at others farther away—brought on by bureaucratic reputation.^20^ What is more, if some audiences are also organized interests that are given voice through the structural design of agencies, then the consequences of agency autonomy may well be to enhance interest group liberalism (Lowi 1969). Painting audiences with a broad brush sidesteps such “messy” normative issues.

The implications are especially important in a context where public organizations are increasingly found to be relying upon accountability‐seeking behavior, voluntarily embedding themselves in new accountability ties, beyond formal requirements. Customer engagement, stakeholder involvement, and “collaborative regulation” are the *noms du jour* in regulatory practice (Heims and Lodge 2018). A whole host of bureaucratic agencies in the US and European contexts are found to engage in accountability‐seeking behavior (e.g., Busuioc 2010; Karsten 2015; Koop 2014; Magill 2009; Reiss 2011) as well as a variety of stakeholder engagement and entrepreneurial activities (Arras and Braun 2018; Busuioc and Jevnaker 2020; Braun and Busuioc 2020; Wood 2018). In this context, it becomes increasingly important to study the extent to which these new practices enhance or detract from existing formal controls.

## Conclusion: With Great Power Comes Great Responsibility?

We have reflected on the normative implications of a reputational account of administrative power for political control and bureaucratic responsiveness, exploring the pitfalls of reputation. We have done so precisely because the reputation literature itself has not. To the extent that reputations are cultivated by benevolent actors acting in the public interest to galvanize support for popular policies and to legitimate their existence, a positive view of reputation is appropriate. It is important to recognize, however, that like all power, RSA can be used responsibly or abused. Taking bureaucrats seriously as political actors requires that the abuse of RSA be given serious consideration. In the hands of strategic actors, powerful reputations can be deployed to tie the hands of legitimate political principals, to deflect oversight of agency action, and to disguise—at least for some time—regulatory failures. In fact, a growing number of empirical studies are reporting suboptimal dynamics associated with aspects of reputation‐building (e.g., Busuioc 2016; Carpenter 2002; Etienne 2015; Maor, Gilad, and Bloom 2013; Maor and Sulitzeanu‐Kenan 2013; Moynihan 2012). RSA becomes especially problematic in this context because it blunts incentives for monitoring and oversight.

A reputational perspective reveals the potential for accountability deficits of the bureaucracy at their most problematic. In a context of diminished oversight, where RSA is the product of undeserved reputation and bureaucratic claims‐making is cleverly done, our elected representatives risk being none the wiser. In the case of an agency held in high regard by audiences, oversight brings political costs imposed by audiences themselves, and this, in turn, diminishes principals’ incentives for monitoring. Moreover, when an agency is perceived as having a strong reputation, audiences are less likely to sound fire alarms, which also compounds the principal's information problem. What is more, even if an agency's image is closely correlated with its actual capacity, reputation need not have been derived in a democratic or representative way. A diverse audience does not a representative audience make.

We call for more research into (1) the impact of reputation on political control and practices of democratic accountability, (2) the role of audiences in this respect, (3) the composition and representativeness of coalitions of esteem, and (4) the impact of informal reputation‐building on the structure and strategy of political control as well as how these might vary across institutional contexts or task environments (see Boon, Salomonsen, and Verhoest 2019). Reputation‐building as a practice of legitimation is often discussed in terms of its positive effects (e.g., Moffitt 2010, 2014), or as an additional obligation that organizations take on to mitigate or to remedy deficits owing to existing hierarchical arrangements (Schillemans 2008). Yet our argument suggests that the relationship between formal and informal controls as legitimizing devices might be an inverse one. On this view, it becomes increasingly important to study the impact of informal authority on the use of formal controls.

There are “pathologies” of public administration under systems of representative government that are important to consider. On the one hand, Fox and Jordan (2011, 843–44), argue that electoral accountability is undermined by delegation when three conditions are met: (1) politicians have more information than the voters about what a bureaucracy will actually do if they receive a delegation, (2) bureaucrats’ expertise is sufficiently real to make a potentially harmful condition for a politician's electoral constituency disappear, and (3) politicians' electoral motivation does not entirely overtake their policy interests. When all are satisfied, voters cannot distinguish delegations that are beneficial to or costly for them, and well‐intentioned politicians are willing to mask the truth about benefits and costs from them. On the other hand, a high‐accountability “spoils system” does not raise the quality of public administration (Lewis 2007). These kinds of problems may lead to arguments that RSA is worth the risk to democracy. This is fine as long as these risks are carefully considered, and we do not think that this has been the case to date.

While democratic accountability, as we have acknowledged, is by no means without its flaws and is clearly open for its own pathologies, it is interesting to consider our argument in the context of the populist challenge to representative government. Populists do not respect existing institutions or norms, and therefore are unlikely to embrace RSA.^21^ In fact, populists get credit from the audiences they court precisely for being anti‐establishment, that is, for disregarding established authority. Without respect for such authority, it is unlikely that RSA can have the benefits that scholars have attributed to it.^22^ What RSA leaves in place is only the possibility of building more RSA, which may be democracy‐preserving, but this depends on the composition of audiences that shifts as populist governments flow and ebb. Moreover, it is important to remember in this context that RSA is explicitly forged among politically mobilized, and not necessarily representative, publics, which are able to exercise concerted political pressure. In other words, it is not a model of bureaucratic insulation from political forces but one of responsiveness to broad publics that need not be broadly representative that can work against “hard wired” and narrow interests. It is unlikely, we think, that reputation will provide the answers to these conundrums. Instead, it has the potential to render them more perplexing.

## Funding

Madalina Busuioc acknowledges funding received for this project from the European Research Council (ERC) under the European Union's Horizon 2020 research and innovation program (grant agreement No. 716439).

## Notes

^ 1^. Our scope conditions restrict us to representative governments, which are in place in much of the developed and developing world. The scope of our argument also excludes non‐democracies.

^ 2.^ The two concepts (expertise and capacity) are closely related, and for our purposes we are loose about the distinction (see also Gailmard and Patty 2007 for a similar approach). For a discussion of the subtle relationship between expertise and capacity however, see Gailmard and Patty (2012, 373–374).

^ 3^. Agency independence that is meant as a credible commitment to a particular policy, as with an independent central bank, is meant to constrain temporally inconsistent preferences on the part of politicians.

^ 4^. Bertelli (2012) argues that this can be a particular problem when developing relational contracts for services with non‐profit or private organizations, and Mason and Fiocco (2017) show evidence of this in the context of immigration.

^ 5.^ For this reason, Carpenter's (2000, 2001) autonomy‐forging behavior relates to strategic capacity‐building as it, too, focuses on this trade‐off.

^ 6.^ From Christiano's (1996) view of representation, influence over the policy agenda influences not the means, but the aims of policy, which are for the people and their political agents to determine. For this reason, we argue that strategic capacity‐building is problematic.

^ 7.^ Miller and Whitford (2016, 154) exemplifies the epistemic view in its delegation‐to‐professionals argument. Policymaking, they contend, involves a community outside the agency: “…the individual professional is monitored and constrained by other professionals, who are enforcing professional norms and holding up professional standards.” What maintains accountable autonomy is the professionalism in Friedrich (1940) and an institutional “stalemate” that “binds subordinate bureaucrats to a cooperative exchange of effort for autonomy” (232) that can override principals’ preferences when they are not for “correct” policies.

^ 8.^ This view contrasts, for instance, with Landemore's (2017, 252) epistemic claim that the cognitive diversity of the many and regular turnover is superior to that of an elite few.

^ 9.^ Our argument is not at odds with stewardship understandings (see Van Slyke 2007; Schillemans 2013). Representative government does not discount that bureaucratic actors can be pro‐socially motivated, and entrusts the *means* of policymaking to bureaucratic agents, while policy *aims* cannot legitimately be agent‐determined (e.g., Christiano 1996).

^10.^ Of course, this may benefit accountability if it reveals that the political principal is acting against deserved reputation or that its audiences are indeed representative.

^11.^ While our argument is focused on political accountability, even when accounting for third‐party accountability forums, the “blunting” effect of RSA on political control holds. Audit bodies, ombudsmen, and courts of audit often lack sanctioning powers of their own. These organizations are not self‐starting, and their findings and recommendations *must* be taken up at the political level. Our observations as to the reduced willingness and motivation of political principals to undertake oversight activity when faced with RSA would apply.

^12.^ The picture is certainly more complex when moving from a monolithic bureaucracy and its reputation (see Maor 2015, 2016). Internal dissent and disagreement over what the organization “stands for” could well result in fire alarms being sounded from within. Our argument is simply that even in such scenarios, RSA would weaken legislators’ motivation to engage in oversight given its low payoffs, resulting in a reluctance to take up such considerations.

^13.^ Misperception can also take the form of undeserved negative perceptions of the bureaucracy (see, e.g., Lerman 2019). We focus on the undeserved reputation scenario as this is the problematic one from our perspective of representative government.

^14.^ An approach to financial regulation that emphasizes the role of principles rather than detailed rules.

^15.^ Supporters ranged from the UK government, the banking industry, to the European Commission (Commissioner McCreevy, Commissioner for Internal Market and Services, stated as late as 2008: “We believe that a ‘light touch’, principle‐based regulation is the best approach for the financial sector.”), to the US Treasury Secretary Hank Paulson (see Black 2008, p. 13).

^16.^
https://www.theguardian.com/business/2013/mar/24/farewell‐fsa‐bleak‐legacy‐light‐touch‐regulator

^17.^ Moffitt (2010, 889) writes, “[r]ather than contribute to drug stability, advisory committee reviews are associated with a greater likelihood of subsequent drug safety label revisions.”

^18.^ Potter's analysis reveals, for instance, that agencies manipulate the comment period when the agency is not ideologically aligned with the president by increasing the number of days a rule is open for comment when interest groups are “in the agency's corner” (122).

^19.^ Exemplifying the positive perspective on reputation, Krause and Corder (2007) put the opposite gloss on their evidence about the insulation of agency's macroeconomic forecasting from political conditions.

^20.^ This comes close to what Dohler (2018) has diagnosed as “implicit coalitional drift”—in the context of political‐bureaucratic relations in Germany: “The implicit version (…) occurs if interest groups, the media or other stakeholders pressure a political principal. In this case, the political principal can drift, i.e. join the new coalition, but is not forced to do so” (3). In Dohler's empirical account however, the coalitional drift is against, rather than on behalf of/benefitting, the bureaucratic agent.

^21.^ We think of populists from the perspective of retrospective accountability rather than as advocates of have‐your‐cake‐and‐eat‐it‐too policies, which any politician could more or less champion. Populists in our view claim mandate representation upon winning one election, but find retrospective accountability less appealing in the next election.

^22.^ In the final chapter of *Reputation and Power*, Daniel Carpenter documents the weakening authority of the FDA in the face of the rise of conservative politics, further underscoring the political underpinnings of RSA. See Miller (2010) on this precise point.
